# A Protective Vaccine against Johne’s Disease in Cattle

**DOI:** 10.3390/microorganisms8091427

**Published:** 2020-09-17

**Authors:** Yashdeep Phanse, Chia-Wei Wu, Amanda J. Venturino, Chungyi Hansen, Kathryn Nelson, Scott R Broderick, Howard Steinberg, Adel M. Talaat

**Affiliations:** 1 Pan Genome Systems, Madison, WI 53719, USA; phanse@pangenosys.com; 2 Department of Pathobiological Sciences, University of Wisconsin-Madison, Madison, WI 53706, USA; chiaweiwu@wisc.edu (C.-W.W.); ajm69@cornell.edu (A.J.V.); chungyi.hansen@wisc.edu (C.H.); howard.steinberg@wisc.edu (H.S.); 3 Research Animal Resources Center, University of Wisconsin-Madison, Madison, WI 53706, USA; knelson@rarc.wisc.edu; 4 Department of Materials Design and Innovation, University at Buffalo, State University of New York, New York, NY 14260, USA; scottbro@buffalo.edu

**Keywords:** live attenuated vaccine, *Mycobacterium avium* subsp. *paratuberculosis*, Johne’s disease, bovine vaccine

## Abstract

Johne’s disease (JD) caused by *Mycobacterium avium* subsp. *paratuberculosis* (*M. paratuberculosis*) is a chronic infection characterized by the development of granulomatous enteritis in wild and domesticated ruminants. It is one of the most significant livestock diseases not only in the USA but also globally, accounting for USD 200–500 million losses annually for the USA alone with potential link to cases of Crohn’s disease in humans. Developing safe and protective vaccines is of a paramount importance for JD control in dairy cows. The current study evaluated the safety, immunity and protective efficacy of a novel live attenuated vaccine (LAV) candidate with and without an adjuvant in comparison to an inactivated vaccine. Results indicated that the LAV, irrespective of the adjuvant presence, induced robust T cell immune responses indicated by proinflammatory cytokine production such as IFN-γ, IFN-α, TNF-α and IL-17 as well as strong response to intradermal skin test against *M. paratuberculosis* antigens. Furthermore, the LAV was safe with minimal tissue pathology. Finally, calves vaccinated with adjuvanted LAV did not shed *M. paratuberculosis* post-challenge, a much-desired characteristic of an effective vaccine against JD. Together, this data suggests a strong potential of testing LAV in field trials to curb JD in dairy herds.

## 1. Introduction

Johne’s disease (JD), caused by *Mycobacterium avium* subsp. *paratuberculosis* (*M. paratuberculosis*), is a chronic infection in ruminants [1] with potential link to Crohn’s disease in humans [2]. Persistent diarrhea and enteritis are hallmarks of JD resulting in significant economic loss due to reduction in milk yield and premature culling. In the USA alone, the dairy industry suffers a loss of USD ~250 million annually [3]. The primary route of transmission of *M. paratuberculosis* is ingestion of fecal material from clinically or sub-clinically infected animals by newborn calves. Clinically infected cows can shed 10^6^–10^8^ CFU/gm of *M. paratuberculosis* in their feces, which can easily spread the infection to new calves with an infectious dose of 10^3^ CFU/animal [4]. It is estimated that 90% of US herds are infected with JD and thus control strategies that prevent fecal shedding are desperately needed to prevent further spreading of the disease [5]. Currently, commercially available vaccines are based on whole cell killed *M. paratuberculosis* and have several drawbacks including interference with a commonly used diagnostic test for bovine tuberculosis and adverse reactogenicity at the injection site usually manifested as a granuloma. Most importantly, the foundation for any successful JD control program is reducing fecal shedding of *M. paratuberculosis* but unfortunately, the available inactivated vaccines have minimally reduced *M. paratuberculosis* shedding [6,7,8,9,10,11]. Clearly, there is a need to develop better vaccines that are safe, effective and compatible with the principle of the differentiation of infected from vaccinated animals (DIVA). Characterization of such vaccine candidates is the subject of this report.

Alternatives to the inactivated vaccines include subunit vaccines that use specific immunogenic antigens [12,13], however, the high cost associated with antigen production and their low efficacy in ruminants [14,15] impede their field application. On the other hand, live attenuated vaccines (LAV) can provide a viable strategy to overcome the aforementioned challenges associated with killed or subunit vaccines and provide benefits such as cost effective manufacturing and induction of a robust cellular immune response [16,17,18,19,20]. Recently, isogenic mutants of virulent strains of *M. paratuberculosis* were used to develop LAV targeting specific auxotroph (e.g., Δ*leuD* [18]) or important virulence factors (e.g., *sigH, lipN* [21,22]). Previously, we used large scale transcriptome analysis to identify activated genes in *M. paratuberculosis* shed from infected cows. This work identified a significantly upregulated virulence gene involved in fatty acid degradation, named lipase/esterase N, (lipN) [22]. Interestingly, some of these mutants were protective against paratuberculosis in both mice [19] and goat [7] models but have yet to be tested in, cattle. For example, a deletion mutant of *M. paratuberculosis K-10::lipN* (pgsN) had a significantly lower colonization and reduced histopathological lesions in mice compared to the wild type *M. paratuberculosis* [22]. Furthermore, in goats, pgsN provided enhanced protective efficacy against a virulent *M. paratuberculosis* challenge as shown by elimination of fecal shedding, reduced histopathological lesion and tissue colonization [7].

In the current study, we tested and compared the efficacy of the LAV to that of the inactivated vaccine (Mycopar^®^, Boehringer Ingelheim Vetmedica, Inc., St. Joseph, MO, USA) by evaluating the generated immune response, safety, tissue colonization, histopathology and fecal shedding following challenge with wild type *M. paratuberculosis.* Further, we evaluated the nature of the vaccine induced immune responses in the presence or absence of the Quil-A adjuvant. Quil-A is a natural saponin derived from the bark of *Quillaja saponaria* tree and is approved as an adjuvant for animal vaccines [23]. Finally, the DIVA capability of the LAV was tested when we applied the bovine tuberculosis comparative intradermal skin test.

## 2. Materials and Methods

### 2.1. Animals

Twenty-eight bull calves were obtained from the Johne’s disease-free University of Wisconsin Dairy Teaching Herd at 3 weeks of age. Serum samples from pregnant dams were screened for *M. paratuberculosis* exposure using the ELISA IDEXX *M. paratuberculosis* Ab Test (IDEXX, Westbrook, ME, USA) performed by the Wisconsin Veterinary Diagnostic Lab (Madison, WI, USA). Bull calves from ELISA negative dams were screened using the same test. Furthermore, environmental fecal samples were randomly collected from each pen and were found to be negative for *M. paratuberculosis* by culture and IS900 PCR. All animal care and experimental procedures were conducted in compliance with the protocols approved by the Institutional Animal Care and Use Committee, University of Wisconsin-Madison protocol #A005070-A01 (3/26/2018). Calves were housed in a biosafety level-2 animal facility in a closed barn with gates separating each group of calves (6/gp). Food was provided ad-libitum. The calves were checked daily for general health.

### 2.2. Vaccine Preparation

The live attenuated vaccine strain (pgsN), was cultured in Middlebrook 7H9 liquid medium (BD Biosciences, Sparks, MD, USA) supplemented with 0.5% glycerol, 2 µg/mL mycobactin J (Allied Monitor, Fayette, MO, USA), and 10% ADC (2% glucose, 5% bovine serum albumin fraction V, and 0.85% NaCl) at 37 °C with gentle shaking. Three to four weeks following incubation (mid log phase culture), bacteria were pelleted by centrifugation at 3200× *g* for 15 min at room temperature in a pre-weighed 50 mL conical tube. After washing, the excess fluid was drained, and an accurate wet weight of the bacterial pellet was determined where 100 mg pelleted wet weight equaled approximately 1 × 10^9^ CFU. The cell pellets were resuspended in sterile PBS at a concentration of 1 × 10^9^ CFU/mL. Each inoculum was split and Quil A (Desert King, San Diego, CA, USA) was added to one portion at a concentration of 500 µg/mL (pgsNQ). The inoculum concentration was confirmed by dilution plating on Middlebrook 7H10 agar supplemented with 0.5% glycerol, 2 µg/mL mycobactin J, and 10% ADC.

### 2.3. Study Design

Calves were randomly assigned based on their age and availability to 4 experimental treatment groups (*n* = 6) and a 5th unchallenged group (*n* = 4) (Supplementary Information Figure S1). Only bull calves were used because they are more available and the experiment was concluded by 15 months of age. The number of calves were selected based on IFN-γ levels from a previous trial conducted by our group [7]. Sample size calculation was performed in ClinCalc online tool with a power of 80% and a confidence level of 95%. All the animal experimental procedures were performed in the morning starting with Untreated, PBS and then the vaccinated groups. The experimental groups were vaccinated with Mycopar^®^ (Boehringer Ingelheim Vetmedica, Inc., St. Joseph, MO, USA), pgsN plus the adjuvant Quil A (pgsNQ), pgsN alone or sham vaccinated with sterile phosphate-buffered saline (PBS) at 4 weeks of age. Calves in the unchallenged group were housed in a separate facility from the experimental groups to avoid any cross contamination among groups. Calves in all groups (except unchallenged group) were challenged at 60 days post vaccination (DPV) with oral inoculation of a clinical strain of *M. paratuberculosis* JTC 1285 [24] (1 × 10^9^ CFU mixed in 10 mL of calf milk replacer). Each calf was allowed to suckle the inoculum from a syringe each day for 5 consecutive days for a total quantity of 5 × 10^9^ CFU. Blood and fecal samples were collected monthly throughout the duration of the clinical trial for immunological and bacteriological analyses as described below. Intradermal skin tests were performed on all calves at 60 days post vaccination, and at 12 MPC as detailed before [7]. All calves were euthanized at 12 MPC and tissue samples were collected for bacteriological and histological examination, as detailed below. Mycopar^®^ was administered according to the manufacturer’s instructions as a single subcutaneous injection into the left dewlap, approximately one inch above the brisket. All other vaccine constructs (pgsN, pgsNQ and PBS) were administered subcutaneously on the left side of the neck for easy access to this body section of the animal and to be consistent with the administration of the most of animal vaccines. All vaccinations were performed by a registered veterinarian for Mycopar vaccine administration. Blinding was done by generating a code for each of the calves and was disclosed only after complete data analysis.

### 2.4. Body Temperature and Weight

Body temperatures of each calf were taken rectally with a digital thermometer pre-vaccination and 1, 2 and 3 DPV to determine if vaccination resulted in a change in body temperature. Calves were weighed pre vaccination and then at 6 months and 12 months post challenge using a Coburn bodyweight tape.

### 2.5. Fecal Culture

Fecal samples were collected fresh from the rectum of individual calves at 1, 7, 30, and 60 DPV and each month for 12 MPC. Fecal samples were processed as previously described [25] with minor variations to optimize *M. paratuberculosis* recovery. Briefly, 3 g of each fecal sample were homogenized with 30 mL 0.75% hexadecyl pyridinium chloride (HPC; Sigma Aldrich, St. Louis, MO, USA) overnight to reduce non-mycobacterial contaminants. The next day, supernatants were harvested, and the remaining fecal material was centrifuged at 1000× *g* for 15 min. This supernatant was removed and added to the initial supernatant and then centrifuged at 3000× *g*. All the pellets were washed with 10 mL PBS and the final pellet was resuspended in 1 mL PBS. Aliquots (100 µL) were plated onto Middlebrook 7H10 agar supplemented with 0.5% glycerol, 2 µg/mL Mycobactin J, 10% ADC and the following antibiotics: vancomycin (100 µ*g*/mL), amphotericin B (50 µg/mL), and nalidixic acid (100 µg/mL; VAN). Samples were incubated at 37 °C for 3 months. Suspected *M. paratuberculosis* colonies were counted and verified with Ziehl–Neelsen staining. An additional 100 µL aliquot was taken for analysis by PCR to detect IS900, a MAP-specific target. DNA was isolated from this sample using a ZR Fecal DNA MiniPrep kit (Zymo Research, Irvine, CA, USA) following the manufacturer’s instructions but using BioSpec 0.1 mm Zirconia/Silica beads and a BioSpec Mini-Beadbeater-16 (BioSpec Products, Bartlesville, OK, USA) for 2 min to homogenize the samples. PCR was used to amplify IS900 [25].

Saliva was collected from each calf 1, 7, 30 and 60 DPV using a sterile foam swab (Item 71-4501, Swab-Its, Springfield, MA, USA) rubbed along the tongue until saturated. The swab was inserted into a 15 mL tube and 2 mL PBS was added. The tubes were vortexed for approximately 20 s and 1 mL PBS was removed from each tube and added to a clean, sterile microcentrifuge tube. Aliquots (100 µL) were plated onto 7H10 agar medium as above or boiled for 10 min in a heat block. A 5 µL aliquot of the boiled saliva sample was used for PCR to detect IS900.

### 2.6. Necropsy, Tissue Collection and Culturing

Calves were euthanized at 12 MPC and tissues were collected for bacterial culture and histology. Euthanasia was performed by a licensed veterinarian using American Veterinary Medical Association approved commercial euthanasia solution (sodium pentobarbital) following recommended dose (>100 mg/kg) for all experimental treatment groups. Death was confirmed by lack of heartbeat, respiration and lack of corneal reflex. All organs for each calf were examined for macroscopic pathological changes at necropsy. The following tissue specimens were collected and processed for histological examination: duodenum, duodenal lymph nodes, 8 sections from proximal to distal jejunum, 8 jejunal lymph nodes, 3 sections of ileum, ileal lymph nodes (ILN), ileocecal valve, cecum, spiral colon, liver, hepatic lymph node, spleen, and the prescapular lymph node ipsilateral to the injection sight. Tissue specimens (3 × 1 cm^2^) were fixed in 10% buffered formalin, embedded in paraffin blocks and stained with hematoxylin and eosin (H&E) or acid fast staining [26]. All tissue slides were examined by a board-certified pathologist (on blind-basis) and lesions were given a severity score based on the description of gross and microscopic lesions [24]. For the jejunum and ileum, where multiple sections were taken, the 3rd section from each animal were taken for histology and culturing. For culturing, the mucosal lining of the entire intestinal samples was scraped with a glass slide and 2 g were collected in a Whirl-Pak bag with 10 mL of 0.75% hexadecyl pyridinium chloride (HPC) to disinfect the intestines. Lymph nodes and selected sections from internal organs were cut into small pieces and 2 g were weighed into a Whirl-Pak bag with 10 mL of PBS. All samples were homogenized using a mechanical stomacher (Seward USA, Davie, FL, USA) on high for 10 min. Intestinal samples in 0.75% HPC incubated at room temperature for 4 h. The supernatant was collected in a 50 mL conical tube and centrifuged at 900× *g* for 20 min at room temperature. The supernatant was poured off and the pellet was resuspended in 1 mL of 7H9 enriched with VAN. Samples were incubated overnight at 37 °C. The next day 200 µL of each sample and a 1:10 dilution were plated onto 7H10 Middlebrook agar supplemented with 0.5% glycerol, 2 µg/mL mycobactin J, 10% ADC, and VAN. An additional 200 µL was dispensed onto Harrold’s Egg Yolk medium (HEYM) slants enriched with VAN. Each sample was incubated at 37 °C for 12 weeks. Colonies of *M. paratuberculosis* were counted, and suspected colonies were confirmed by Ziehl-Neelsen staining and in a few cases, genotyped with PCR [27].

### 2.7. Antibody Response

Whole blood was collected in EDTA vacutainer tubes (Becton Dickinson and Co., Franklin Lakes, NJ, USA) by the jugular vein from each calf monthly. A 0.5 mL aliquot was allowed to clot at room temperature. The sample was centrifuged at 1000× *g* for 10 min at room temperature. The serum was collected and stored at −20 °C. The serum was evaluated for the presence of antibodies to *M. paratuberculosis* using Parachek^®^ 2 (Prionics USA, Inc. Omaha, NE, USA) following manufacturer’s instructions.

### 2.8. Intradermal Skin Test

At 60 DPV and 12 MPC, we performed an intradermal skin test on all calves using standard purified protein derivatives (PPD) from *M. avium, M. bovis,* and *M. paratuberculosis* (Johnin) obtained from the National Veterinary Services Laboratory (Ames, IA, USA). A region on the side of the neck was clipped and the skin thickness was pre-measured using digital calipers 0.1 mL of each PPD were injected into the intradermal layer of the skin. The response to the PPD injection was measured at 0 and 72 h post-injection. The values obtained at 72 h were subtracted from 0 h values and presented as change in skin thickness.

### 2.9. IFN-γ Release Assay

Peripheral blood mononuclear cells (PBMCs) were isolated from each calf monthly as previously described [15]. Briefly, 12–15 mL of whole blood was collected from the jugular vein in EDTA vacutainer tubes (Becton Dickinson and Co., Franklin Lakes, NJ, USA). The blood was diluted in a 1:1 ratio with HYCLONE RPMI-1640 (GE Life Sciences, Logan, UT, USA) and mixed by inversion. The mixture was carefully layered onto 7–10 mL of Histopaque^®^-1077 (Sigma-Aldrich, St. Louis, MO, USA) in a 50 mL conical tube. The PBMCs were separated by differential centrifugation at 400× *g* for 30 min at room temperature with no brake. The PBMCs were removed and washed twice with RPMI-1640 by centrifugation at 300× *g* for 10 min at 4 °C. The pellet was resuspended in 1 mL of lysis buffer (1:10 dilution of 0.17 M Tris-HCl to 0.16 M NH_4_Cl) for 2–5 min to lyse any remaining erythrocytes. RPMI-1640 was added for a final wash, and the resulting pellet was resuspended in enriched medium RPMI-1640 with 2 mL L-Glutamine, 10% heat inactivated fetal bovine serum (FBS), 1× non-essential amino acids (Gibco MEM NEAA 100x Thermo Fisher Scientific, Waltham, MA, USA), penicillin (100 IU/mL) and streptomycin (100 µg/mL)). The PBMCs were aliquoted 1 × 10^6^ cells per well and stimulated with 10 µg/mL Johnin PPD or unstimulated for 72 h. The supernatants were separated from the PBMCs and frozen at −80 °C. The IFN-γ production was measured in culture supernatants using a monoclonal antibody-based sandwich ELISA (Bovigam^®^, Thermo Fisher Scientific, Waltham, MA, USA), as per manufacturer’s instructions after samples were diluted 1:10 due to over absorption of the antibodies. The ELISA plates were developed until the OD 650 nm of the positive control was approximately 0.35. Following addition of the stop solution, plates were read at OD 450 nm. The OD results are expressed as ELISA index values by dividing the mean OD of the PPD-stimulated supernatant by the mean OD of the corresponding unstimulated supernatant. When needed, supernatants were diluted and multiplied by the dilution factor before an ELISA index value was calculated with the formula described above.

### 2.10. Cytokine Profiling

The frozen cell culture supernatants obtained from either Johnin PPD stimulated or unstimulated PBMCs were evaluated for cytokine and chemokine secretion using a commercially available Quantibody Bovine Cytokine Array kit (Raybiotech, GA) using manufacturer’s instructions. The cytokines evaluated were IFN-γ, IFN-α, IL-13, IL-1α, MIG (CXCL9), and TNF-α. The slides were scanned using Innoscan 710 (Innopsus, France) scanner. Data was analyzed using Q-Analyzer software (Raybiotech, GA, USA). A commercially available bovine IL-17A ELISA kit (Raybiotech, GA, USA) was used following manufacturer’s instructions using 1/10 dilution of cellular supernatants.

### 2.11. Statistical Analysis

A multi-layer, Principal Component Analysis (PCA) was employed to ensure equal weighting of all experimental readout from this study. PCA operates by decomposing a data set into two separate matrices: the scores which describes the relationship of the conditions (in this case, different samples) and the loadings matrix which describes the correlations of the responses (e.g., colony counts, histology scores). In each case, a sufficient amount of information was captured in one dimension (where we define sufficient as capturing over 80% of the variance in the data), and thus a new parameter was developed for each measure, thereby providing a common quantification of the measures and removing bias [28,29,30,31]. Thus, each animal tested had three associated values, corresponding with the three measures (fecal shedding, cytokine levels, granulomatous inflammation score) employed for this study. A fourth PCA was then applied to this new data to develop a scoring of the data, to assess similarity in the data. The multi-layer, PCA provides an important advancement in extracting correlations in data which otherwise have high uncertainty. Of important note for the purpose of the analysis described here is that all these PCA analyses perform a normalization by standard deviation, so that the fourth level PCA analysis is based on input data that can be compared. Differences between the groups were analyzed using Student’s *t* test. A probability value of less than 0.05 was considered significant for all tests. All statistical analysis was performed using GraphPad Prism 6 (GraphPad Software, Inc., La Jolla, CA, USA).

## 3. Results

### 3.1. Safety of Live Attenuated Vaccine Candidates

**Figure 1:**
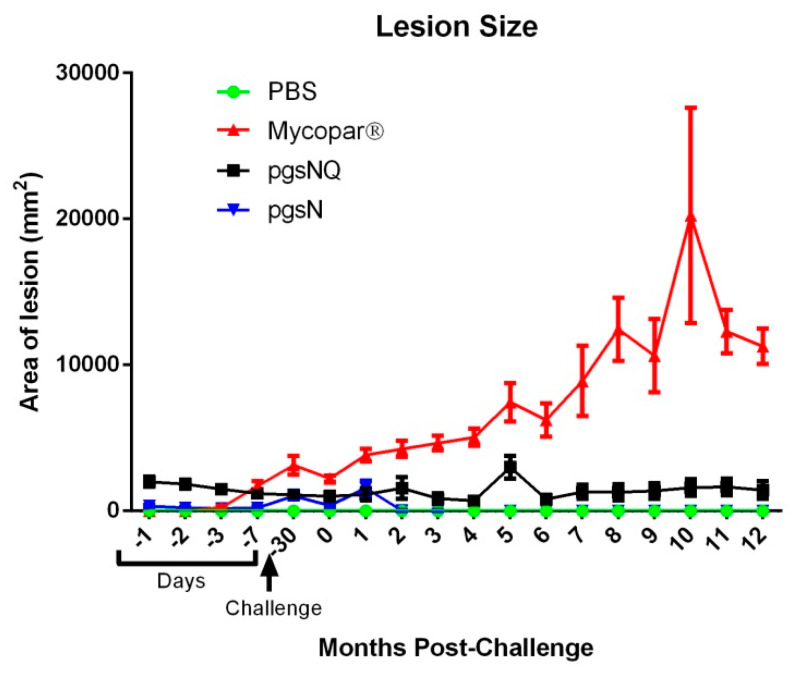
Safety of vaccine candidates in calves. Vaccine injection site lesions were measured throughout the duration of the study. Data are expressed as arithmetic means, with the error bars representing the standard deviation.

As expected, all the calves survived through the course of the study following 2 months of vaccination and of 12 months of follow up analyses. The body temperatures of the vaccinated calves were in the normal range (38–40 °C) at 1 and up to 3 DPV and was not statistically different than the PBS group calves Figure S2 in Supplementary Materials. The calves vaccinated with live attenuated or inactivated vaccines had weight gain comparable to that of PBS vaccinated calves indicating that all vaccines used did not cause significant changes in overall body conditions (Figure S3). However, calves vaccinated with Mycopar (Figure 1), displayed an increase in skin indurations at the site of injection. The average lesion size gradually increased after the challenge in all calves in the Mycopar vaccine group. In contrast, the same skin measurements to the pgsN and pgsNQ calves were minimal and comparable to the PBS vaccinated calves. None of the fecal samples from calves of any treatment group were positive for *M. paratuberculosis* as tested by culture and PCR. No *M. paratuberculosis* was observed in the saliva samples of pgsN group by culturing, and minimal by PCR (Table S1 and S2), indicating presence of bacterial DNA. In summary, while Mycopar vaccination was associated with injection site lesions, both the pgsN and pgsNQ vaccine candidates were more safe and well-tolerated by calves.

### 3.2. Live Vaccines Reduced Key Parameters of Johne’s Disease

None of the calves from the unchallenged group shed any *M. paratuberculosis* at any of the times during the study, confirming the JD free status of the experimental calves. At 10 months post challenge, 50% of the control calves vaccinated with PBS shed mild (1–3 CFU) to medium (5–9 CFU) of *M. paratuberculosis* whereas, 33% of calves from the Mycopar group had mild shedding (Table 1). Further, one animal in the pgsNQ group shed at 11 MPC. AT 12 MPC, one calf in the PBS group had mild shedding. In contrast, none of the calves vaccinated with pgsN shed detectable levels of *M. paratuberculosis* throughout the course of the study.

*M. paratuberculosis* colonies were not detectable in calves belonging to the vaccine or control groups. To verify the culture negative status, we also selected representative tissues (*n* = 5) from two calves in the PBS control group (with detectable fecal shedding of *M. paratuberculosis*) using the Mycobacterial Growth Indicator Tube (MGIT) detection system. No growth was observed in any of the PBS-control tissues except for the jejunum of one of the calves, an indication of an overall very low level of tissue colonization using the challenge protocol and strain in this experiment.

**Figure 2:**
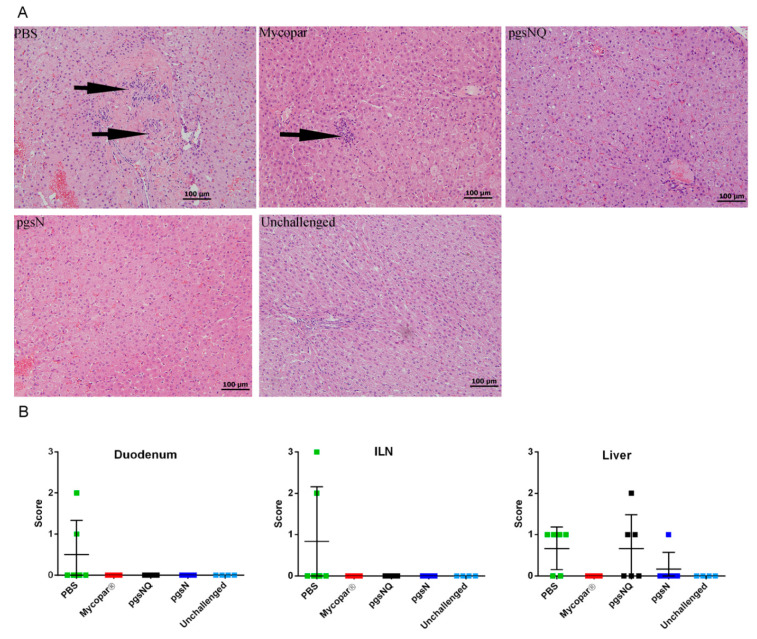
Histopathology of tissues from vaccinated calves following challenge with *M. paratuberculosis* JTC 1285. (**A**) Liver sections stained with hematoxylin and eosin collected from calves vaccinated with phosphate-buffered saline (PBS), Mycopar, pgsNQ, pgsN and unchallenged at 12 MPC. Arrows indicate granuloma infiltrates. (**B**) Granulomatous inflammation scores in duodenum, ileal lymph node (ILN) and liver at 12 MPC. Data are expressed as mean values with error bars representing the standard deviation. Individual calves are indicated by solid box.

Finally, more than 25 tissue samples per animal were collected for a detailed histopathological analysis. In the unchallenged, inflammation or lesion associated with JD were not observed in any of the gut, associated lymph node or liver sections (Figure 2A). In the PBS and challenged calves, granulomatous inflammation was present in most sections of the gastrointestinal (GI) tract, consistent with *M. paratuberculosis* infection. These calves also showed substantial mixed inflammation (lymphocytic, plasma cellular, neutrophilic and eosinophilic) in all sections of the GI tract. Moreover, substantial reactive lymphoids and hyperplasia of Gut Associated Lymphoid Tissue (GALT) were prominent in most GI sections. Lymphohistiocytic granulomatous inflammation in 4/6 livers was also noted. In Mycopar and pgsNQ vaccinated calves, lymphoplasma cellular inflammation was noticeable with a minimal neutrophilic and eosinophilic component and moderate reactive lymphoid and GALT hyperplasia. Mycopar group had the mildest liver reaction amongst the vaccinated groups. Interestingly, pgsN vaccinated calves showed not only the least inflammatory response but also of mildest intensity amongst all vaccine groups (Figure 2B). In general, both pgsN and pgsNQ groups displayed the least level of fecal shedding and were associated with the lowest tissue lesion scores.

### 3.3. Robust Cellular Immunity Generated by Live Vaccine Candidates

**Figure 3:**
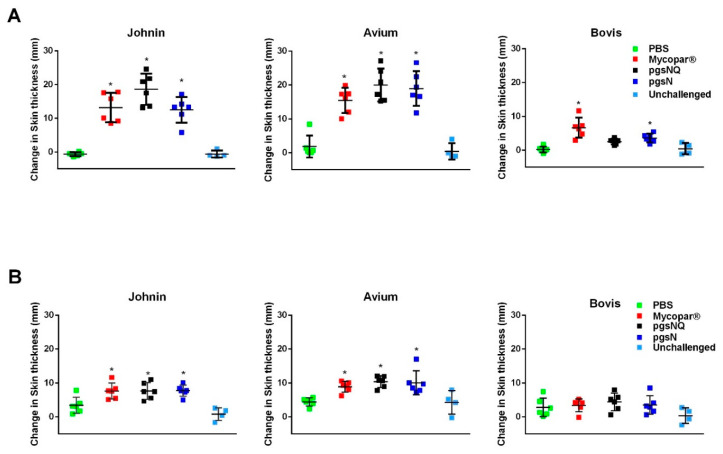
Vaccine induced delayed type hypersensitivity in calf groups. Intradermal skin test performed on calves at 2 months post vaccination (**A**), and 12 months post challenge (**B**). Each graph shows the difference of pre injection skin thickness measurement (0 h) from the skin thickness measurement 72 h after purified protein derivatives (PPD) injection. Each data point represents an individual animal, while the horizontal lines indicate the mean value for each group with error bars representing the standard deviation; * *p* < 0.05 compared to the PBS group. The 0 h skin thickness was subtracted from the 72-h skin thickness prior to statistical analysis.

At 2 MPV, all calves vaccinated with Mycopar, pgsN and pgsNQ showed significant skin induration (compared to PBS group) in response to Johnin PPD at the time of reading (72 h post injection) (Figure 3). However, pgsNQ vaccinated calves had the highest skin induration among all groups. Importantly, the same calves belong to pgsNQ group did not respond significantly to *M. bovis* PPD. In contrast, the Mycopar and pgsN vaccinated calves had significant skin induration against *M. bovis* PPD, suggesting a potential problem with using the skin test to differentiate infected from vaccinated calves with Mycopar or pgsN at a given time point. No significant skin induration was observed in calves from the PBS or unchallenged group. Similarly, all vaccinated calves regardless of the vaccine used exhibited a significant skin induration against Johnin at 12 MPC. However, at this time point, none of the vaccinated calves showed any skin reactivity against *M. bovis* PPD (Figure 3B). Because of the similarity between *M. paratuberculosis* and *M. avium,* all vaccinated calves reacted significantly to *M. avium* PPD at both 2 MPV and 12 MPC.

### 3.4. Cytokine and Chemokine Responses to LAV Candidates

**Figure 4:**
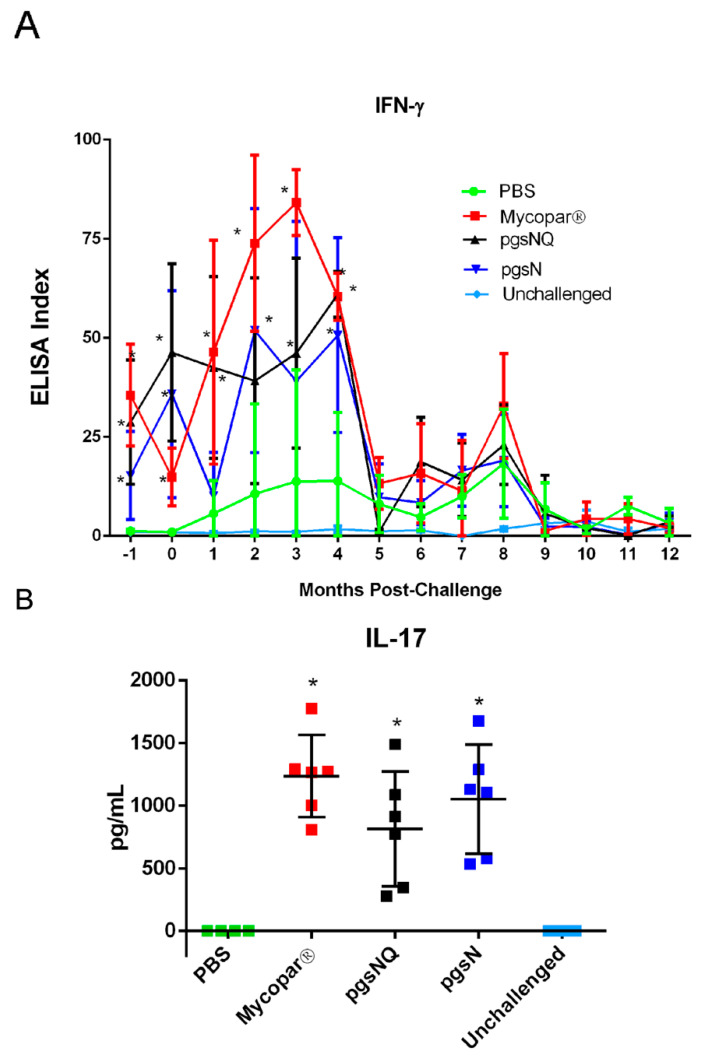
Blood levels of key cytokines in vaccinated calf groups. (**A**) IFN-γ response in calves before vaccination (0, −1 month), post vaccination (1, 2 months) and post challenge (2–12 months). Peripheral blood mononuclear cells (PBMCs) were isolated from whole blood and stimulated with Johnin PPD for 72 h. IFN-γ levels in culture supernatants were determined using a Bovigam ELISA kit. Data are expressed as an ELISA index, with error bars representing the standard deviation; * *p* < 0.05 compared to the PBS group. (**B**) IL-17 response in calves 2 months post vaccination. PBMCs were isolated from whole blood and stimulated with Johnin PPD for 72 h. IL-17 levels in culture supernatants were determined using an ELISA kit. Data are expressed as mean values with error bars representing the standard deviation; * *p*< 0.05 compared to the PBS group.

In *M. paratuberculosis*-stimulated PBMCs, IFN-γ was significantly induced in the pgsN, pgsNQ and the Mycopar vaccinated calves at 2 MPV (Pre-challenge) compared to the PBS group (Figure 4A). It is noteworthy to mention here that the IFN-γ response from pgsNQ group remained significantly higher than PBS groups till 4 MPC (*p* < 0.05). In addition to a Th1 proinflammatory cytokine response, IL-17 response was also evaluated by quantifying IL-17 levels at 2 MPV. All vaccinated calves (Mycopar, pgsN and pgsNQ) had significantly higher secretion of IL-17 cytokine after stimulation with johnin PPD (*p* < 0.05) (Figure 4B). None of the calves from PBS or unchallenged group had any detectable IL-17 responses.

**Figure 5:**
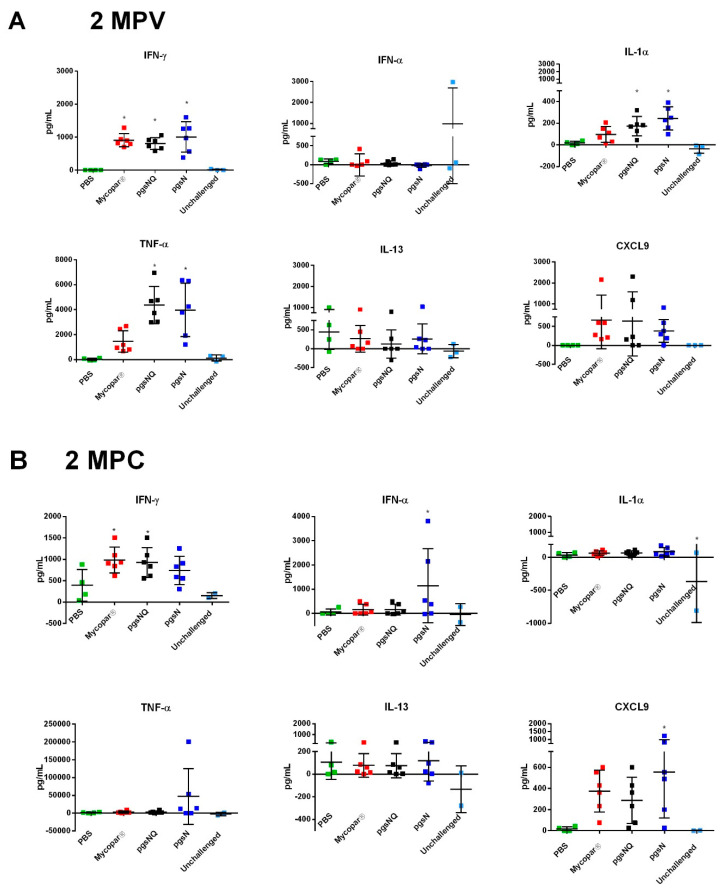
Protein array analysis of key cytokine levels in vaccinated calf groups. PBMC samples were isolated from whole blood and stimulated with Johnin PPD for 72 h. Cytokine levels in culture supernatants were determined using a bovine cytokine array kit at (**A**) 2-months post vaccination (MPV) and (**B**) 2-months post challenge (MPC). Data are expressed as mean with error bars representing standard deviation; * *p* < 0.05 compared to the PBS group.

Furthermore, when multiplex arrays were used to measure multiple cytokines at key points post vaccination and post challenge, calves vaccinated with pgsN and pgsNQ vaccines induced significantly higher responses of IFN-γ, IL-1α and TNF-α at 2 MPV compared to the PBS control group (Figure 5A). Interestingly, Mycopar vaccinated calves exhibited only IFN-γ responses, but no other proinflammatory cytokines. Additionally, the chemokine CXCL9 (MIG) which is IFN-γ dependent was upregulated (a measure of IFN-γ bioactivity) in calves vaccinated with pgsN and pgsNQ vaccines. Importantly, none of the vaccine groups induced significantly higher levels of IL-13 (*p* > 0.05), a Th2/anti-inflammatory cytokine. At 2 months post challenge (MPC), calves vaccinated with pgsN and pgsNQ had higher levels of cytokines IFN-α, and IFN-γ, respectively compared to PBS controls (*p* < 0.05) (Figure 5B). In fact, pgsN vaccinated calves had a ~10 fold increase in TNF-α post challenge. No differences were observed between any of the groups for the anti-inflammatory cytokine IL-13 at 2 MPC. Similar to 2 MPV, pgsN and pgsNQ vaccinated calves had higher secretion of CXCL9 and were significantly higher than PBS controls at 2 MPC (pgsNQ; *p* < 0.05). Overall, the LAVs induced a robust proinflammatory cytokine responses post vaccination as marked by IFN-γ and IL-17 in all vaccine group but maintained and expanded this response (e.g., IFN-α and TNF-α) post challenge only in pgsN vaccine group.

### 3.5. Humoral Immunity Generated by Live Vaccine Candidates

**Figure 6:**
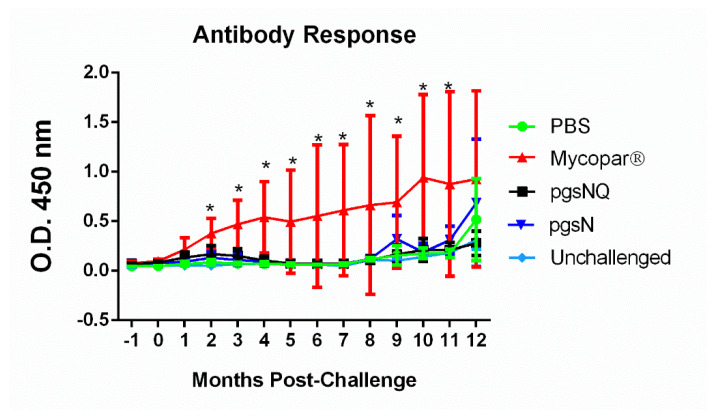
Humoral immunity in vaccinated and challenged calf groups. Commercial ELISA were used to analyze antibody levels in serum samples collected on monthly basis. Error bars represent the standard deviation of the average for each animal group for one month before challenge and 12 months post challenge; * *p* < 0.05 compared to the PBS group.

As expected, Mycopar was the only group that elicited significantly higher levels of IgG from 2 MPC till the termination of the study at 12 MPC (Figure 6). At 12 MPC, calves from PBS, pgsN and pgsNQ also started to develop an antibody response most likely due to the response to the *M. paratuberculosis* infection. Overall, live attenuated vaccines elicited robust cellular immune responses with little induction of humoral immunity.

### 3.6. Composite Analysis of Vaccine Efficacy

**Figure 7:**
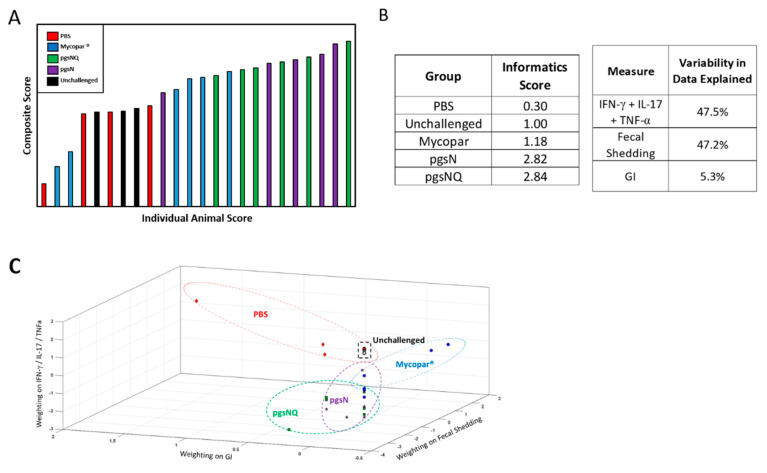
Composite analysis of the efficacy of live and inactivated vaccine candidates. (**A**) Ranked animal scores of the composite analysis based on fecal shedding, histological lesions and cytokine levels using principal component analysis (PCA). (**B**) Group scores with percentage of variability among animal groups using PCA. (**C**) A 3D mapping of calves of individual composite scores based on the readout measures used in the study (cytokine levels, lesion scores and fecal shedding). Note the close proximity of profiles generated by the pgsN and pgsNQ groups compared to Mycopar, unchallenged and infected groups. Each point in (**C**) represents an individual animal.

PCA showed that PBS and Mycopar treatments were largely similar to the unchallenged treatment (Figure 7A,B), while the pgsN and pgsNQ were clustered separately from the PBS treatment. Both parameters of cytokine profiles and fecal shedding counts had a significant impact on the overall composite score while the histological lesions had minimal impact. This was further illustrated by mapping of the composite scores in Figure 7C where the differences in PBS calves were due primarily to granulomatous inflammation scores, while fecal shedding in the Mycopar group calves were different from unchallenged calves. The pgsN and pgsNQ scores were different from other treatments primarily due to the impact of cytokine profiles (e.g., TNF-α). Overall, this analysis shows that pgsN and pgsNQ not only had a desirable impact by reducing the fecal shedding and GI score, but also led to a larger cytokine response.

## 4. Discussion

Vaccination is considered the most effective strategy against JD in the field, mainly due to lack of feasible antibiotic regimens or effective herd management tools to curtail the disease [32]. With the potential link to cases of Crohn’s disease in humans [2,33], it is of a paramount importance to reduce JD prevalence in cattle and *M. paratuberculosis* in dairy products [34,35,36]. Currently, Mycopar^®^ is the only vaccine approved for limited use in the USA [37]. Despite the ability of Mycopar to induce immune responses to JD in calves, shedding of *M. paratuberculosis* is not controlled [38], as confirmed in 33% of the calves in this study, a problem in transmitting the disease to unchallenged calves. In sheep, some animals shed *M. paratuberculosis* and die from multi-bacillary form of JD despite being vaccinated with an inactivated vaccine [39]. In a long-term study of the effect of inactivated vaccine on dairy herds to reduce the transmission of the disease, no significant difference in prevalence was found between vaccinated and non-vaccinated herds [40]. In this report, we assessed the efficacy of the live-attenuated vaccine pgsN (an isogenic mutant of *lipN* gene in *M. paratuberculosis-*K10 background) that has previously shown to induce a robust immune-response and protection against wild type *M. paratuberculosis* in mice and in goats [7,19].

Throughout this study, the licensed vaccine Mycopar^®^ was utilized as a gold standard control, despite its known shortcomings [40,41]. Because of the safety concerns of Mycopar causing granuloma formation at the site of inoculation [42], much attention was given to the safety of vaccine candidates based on pgsN whether QuilA adjuvant was used or not. As expected, calves injected with Mycopar started developing granuloma around seven days post-vaccination that gradually progressed throughout the course of the study with one animal displaying a large sore on the granuloma with purulence at 10 MPC. In contrast, the LAVs exhibited an excellent safety profile and minimal to no lesions at the site of inoculation, corroborating the outcome of applying the same vaccines in goats [7]. The interference of Mycopar with bovine TB diagnostics further makes it a less than ideal vaccine [7]. As indicated in our hands, Mycopar vaccinated calves displayed significant skin indurations after the injection of *M. bovis* PPD clearly showing cross-reactivity to Bovine TB. On the other hand, none of the LAV vaccinated calves developed significant skin indurations against *M. bovis* PPD at 12 MPC, despite mounting a robust reaction to Johnin. In fact, pgsNQ vaccinated calves did not respond to the *M. bovis* PPD at the 2 MPV time point, as well. This lack of reactivity to bovine PPD feature, could be crucial for further developing the LAVs used here as DIVA compliant vaccines (vaccines with a simple assay to differentiate infected from vaccinated animals). The DIVA-compliant vaccines would be very useful to implement to control paratuberculosis in countries where bovine tuberculosis is prevalent and farmers hesitate to use the current JD vaccine. The observed cross reactivity to *M. avium* PPD by calves from all vaccine groups is expected because of the close similarity between *M. avium* and *M. paratuberculosis* but could be employed as a further confirmation of developing immunity against paratuberculosis.

Other live attenuated vaccine candidates included an auxotroph (*M. paratuberculosis* Δ*leuD*) that was attenuated in goats, but induced Th1 proinflammatory and Th17 T cell responses correlated with protective efficacy when used as vaccine against *M. paratuberculosis* challenge [18]. Unfortunately, immunity elicited by such auxotrophs could be short-lived and not robust enough when animals mature. In our hands, the pgsN vaccine induced a quick and robust IFN-γ response within 2–3 months post vaccination that correlated with superior protection against *M. paratuberculosis* challenge in goats [7]. Corroborating the past results, calves vaccinated with LAVs elicited a robust IFN-γ response by 2 MPV. Moreover, a distinct proinflammatory cytokine profile (IL-1α, TNF-α and IFN-γ) was observed from the calves vaccinated with the LAVs both post vaccination (2 MPV) and post challenge (2 MPC). In addition to the proinflammatory cytokines, IL-17 secretion was also upregulated in the LAV vaccinated calves. Because multiple cells (e.g., Th17, γδT or NK cells) can secrete IL-17 [43], which cell type (s) were activated following LAV vaccination remains a question for future studies. In contrast, Mycopar vaccine only elicited the secretion of IFN-γ but not TNF-α and IL-1α cytokines. Interestingly, at 2 MPC inflammatory chemokine CXCL9 was secreted significantly higher in calves vaccinated with pgsN. The chemokine CXCL9 attracts Th1 cells and inhibits the migration of Th2 cells to the site of infection and may thus improve the vaccine efficacy for intracellular infections [44], a desired feature for effective vaccines. Previously, IL-1α has been shown to be upregulated in ileal tissues of cattle infected with *M. paratuberculosis* and may cause toxicity at high levels [45]. However, type I interferon (IFN-α and IFN-β) inhibits the production of interleukin -1 cytokines (IL-1) including IL-α [46]. Here too, the significant upregulation of IL-1α at 2 MPV in the pgsN vaccinated calves was downregulated at 2 MPC with a corresponding increase in IFN-α in the same group of calves, suggesting the activation of mechanisms to control IL-1α levels. Despite not knowing the exact reason behind repression of IL-1α levels, the downregulation maybe a beneficial feature of pgsN-based vaccines. Overall, the array of cytokines and chemokines employed in this study could serve as a good indicator for the type of vaccine-induced immunity with potential to augment other protection parameters (such as fecal shedding and tissue colonization) which take longer times to develop. In future experiments, the host transcriptome of immunized calves could be analyzed to further profile the type of vaccine-induced immunity among different vaccine formulas.

Surprisingly, the addition of the QuilA adjuvant to pgsN (pgsNQ) did not provide clear benefits to boost the type and/or magnitude of secreted cytokines. In fact, at 2 MPC, the unadjuvanted pgsN vaccine had significantly higher levels of IFN-α and CXCL9 and a ~10-fold increase in TNF-α secretion. Unlike Mycopar, the early induction at 2 MPV of IFN-γ, IL-1α, TNF-α, IL-17 and CXCL9 in pgsN and pgsNQ groups may help in controlling early infection with *M. paratuberculosis* and provide more protection against associated pathological lesions as was clearly shown in liver. Outside the GI tract, liver was a very reflective organ to infection with *M. paratuberculosis* as shown before in calves [26,47]. Unfortunately, tissue colonization with *M. paratuberculosis* was not detectable in the used calf model*,* otherwise, this parameter could help us decipher QuilA has a role in protection. As indicated in this study, we were not able to culture *M. paratuberculosis* from collected organs despite the presence of fecal shedding in the control and Mycopar-vaccine groups and presence of histological lesions and the immune profile associated with *M. paratuberculosis* infection. It is possible that the challenge strain of *M. paratuberculosis* JTC1285 was not adapted to calves, despite being a bovine genotype [27]. By 10 MPC, 50% of the control group calves showed *M. paratuberculosis* shedding while 33% of Mycopar shed *M. paratuberculosis*. The delay in fecal shedding was observed in one animal (17% of the calves) with shedding at 11 MPC for the pgsN group while none of the calves in the pgsNQ vaccine group shed any *M. paratuberculosis*, a highly desired feature of any JD vaccine. This again could be attributed to the superior proinflammatory Th1 and Th17 type immune response induced by the LAVs. Another limitation of this study was not all calves in the PBS group (3 out of 6) shed *M. paratuberculosis.* As mentioned earlier, this could be due to the challenge strain JTC 1285 used in this experiment or the lack of sensitivity of fecal culturing technique used here. Even during natural infection, intermittent fecal shedding of *M. paratuberculosis* is a main feature of JD [48].

A key challenge of our comparative study is how to evaluate all experimental readouts of vaccines to select protective candidates for further development. Another complication is the dissimilar format for the used assays (e.g., ELISA titers vs. CFU count). Additionally, fecal shedding is measured at multiple times, while cytokine profiles and histology were measured at a single time point. To address these challenges, we employed mathematical modeling of vaccine parameters to generate a composite score based on principal component analysis (PCA) that was used before to decode complex datasets, in an unbiased manner [49,50,51]. A clear advantage of the PCA is that all the results are based on a multi-level analyses so that the weighting of the measures is not based on a particular parameter, but on explaining the difference among parameters used to evaluate vaccine candidates. Importantly, the calculated composite index showed no impact to outliers but remarkable consistency within each animal group. Therefore, the conclusions drawn from this analysis have minimized any uncertainty associated with individual measures. Strikingly, the composite scores quantitatively showed that both pgsN and pgsNQ vaccine candidates closely achieved the target properties (low shedding and robust immunity) of an ideal vaccine against JD. Together, these data show that the adjuvanted and unadjuvanted pgsN LAV elicited a robust proinflammatory immune response and provided protection against *M. paratuberculosis* challenge. Moreover, the LAVs also exhibited an excellent safety profile with the potential for developing an effective DIVA assay to differentiate vaccinated calves from those infected with *M. bovis*, a further confirmation that these vaccines are promising candidates for field application to control Johne’s disease in dairy cattle and other ruminants such as goats and sheep.

## Figures and Tables

**Table 1:** *M. paratuberculosis* challenge strain fecal shedding.

Treatment Group	Fecal Shedding (CFU/gm) by Months Post Challenge(Mean [% Positive])		
	10	11	12
PBS	10 (50%)	0	3 (17%)
Mycopar	2 (33%)	0	0
pgsN	0	5 (17%)	0
pgsNQ	0	0	0
Unchallenged	0	0	0

A 3 CFU limit of detection was used. *M. paratuberculosis* was not detected from months 1–9.

## Supplementary Materials

The following are available online at https://www.mdpi.com/2076-2607/8/9/1427/s1, Figure S1: Experimental timeline for vaccination and challenge, Figure S2: Body temperature following vaccination, Figure S3: Body weights following challenge. Table S1: Vaccine shedding in saliva and fecal samples detected by culture on solid media, Table S2: Vaccine shedding in saliva and feces detected by IS900 PCR.
