# Supplementary material for: A Protective Vaccine against Johne’s Disease in Cattle
Source: Microorganisms. 2020 Sep 17;8(9):1427. doi: 10.3390/microorganisms8091427 (PMC7564561; doi:10.3390/microorganisms8091427)
Supplement: Supplementary file 1 [file microorganisms-08-01427-s001.pdf]

Table S1. Vaccine shedding in saliva and fecal samples detected by culture on solid media. A + indicates at least one unambiguous positive sample from that group. Bracket indicates number of animals positive /number of animals tested. “0” denotes negative test result per animal tested. N/A denotes samples were not available for this particular time point for the thought that no culturable vaccine could be obtained from the PBS, inactivated vaccine or unchallenged animals. This decision was reversed at later time points.

Saliva

	**1 DPV**	**7 DPV**	**30 DPV**	**60 DPV**
**PBS**	0/6	0/6	0/6	0/6
***pgs*N**	0/6	+(1/6)	+(1/6)	0/6
pgsNQ	0/6	0/6	0/6	0/6
**Mycopar®**	0/6	0/6	0/6	0/6
**Unchallenged**	0/4	0/6	0/4	0/4

Feces

	**1 DPV**	**7 DPV**	**30 DPV**	**60 DPV**
**PBS**	0/6	N/A	0/6	0/6
***pgs*N**	0/6	0/6	0/6	0/6
pgsNQ	0/6	0/6	0/6	0/6
**Mycopar®**	0/6	N/A	0/6	0/6
**Unchallenged**	0/4	N/A	0/4	0/4

Table S2. Vaccine shedding in saliva (top) and feces (bottom) detected by IS900 PCR. A + indicates at least one unambiguous positive sample from that group. Bracket indicates number of animals positive /number of animals tested. "0" denotes negative test result per animal tested. N/A denotes samples were not available for this particular time point for the thought that no culturable vaccine could be obtained from the PBS, inactivated vaccine or naïve animals. This decision was reversed at later time points.

Saliva

	**1 DPV**	**7 DPV**	**30 DPV**	**60 DPV**
**PBS**	0/6	N/A	0/6	0/6
***pgs*N**	+ (1/6)	0/6	0/6	0/6
pgsNQ	+ (2/6)	0/6	+ (1/6)	0/6
**Mycopar®**	0/6	N/A	0/6	0/6
**Unchallenged**	0/6	N/A	0/6	0/6

Feces

	**1 DPV**	**7 DPV**	**30 DPV**	**60 DPV**
**PBS**	0/6	N/A	0/6	0/6
***pgs*N**	0/6	0/6	0/6	0/6
pgsNQ	0/6	0/6	0/6	0/6
**Mycopar®**	0/6	N/A	0/6	0/6
**Unchallenged**	0/6	N/A	0/6	0/6

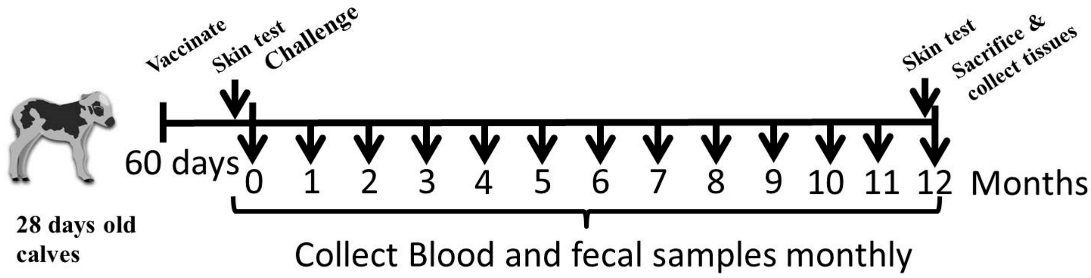

Fig S1. Experimental timeline for vaccination and challenge. Calves, 60 days old, were vaccinated by subcutaneous injection and challenged 60 days later with a virulent strain of *M. paratuberculosis* JTC 1285 by oral inoculation of bacteria in milk.

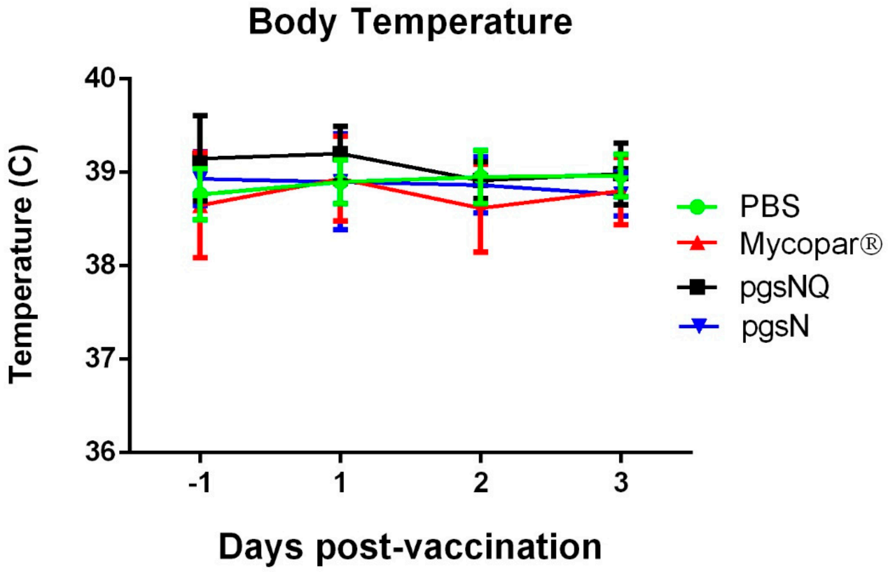

Fig S2. Body temperature following vaccination. Temperature of the calves were measured pre and post vaccination. Data are expressed as arithmetic means, with the error bars representing the standard deviation.

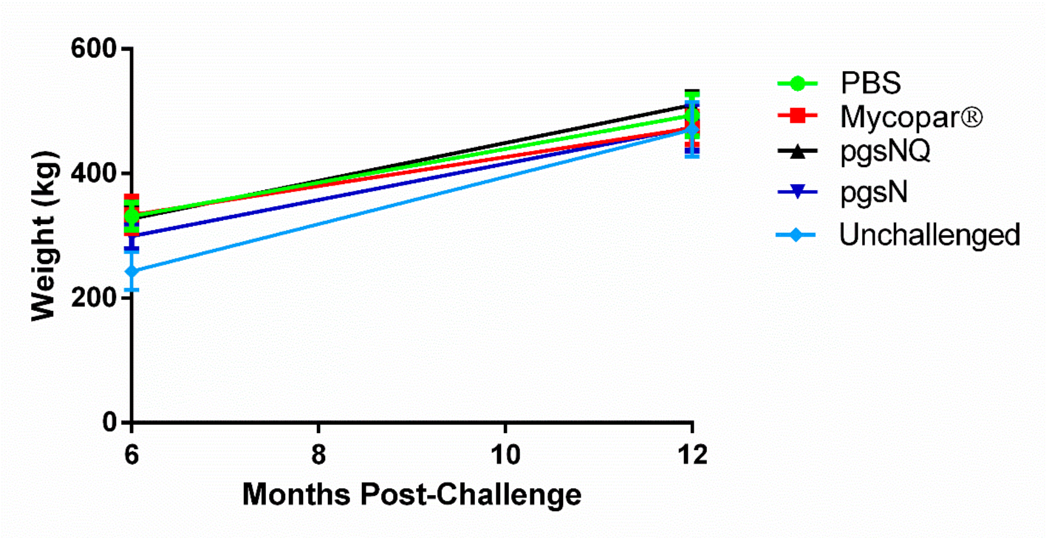

Fig S3. Body weights following challenge. Weights of the calves were measured post challenge. Data are expressed as arithmetic means, with the error bars representing the standard deviation. All calves were weighted ~ 45 kg at time of start of birth.
